# Probiotic supplementation during pregnancy or infancy for the prevention of allergic rhinitis in infants: A systematic review and meta-analysis of Randomized controlled trials^☆^

**DOI:** 10.1016/j.waojou.2025.101124

**Published:** 2025-10-04

**Authors:** Zhengxiu Xiao, Haiqin Luo, Hao Liu, Hui Chen, Jun Bai, Wang Liao, Shi Wu Wen, Daniel Krewski, Huiling Shang, Rihua Xie

**Affiliations:** a School of Nursing, Jinan University, Guangzhou, Guangdong, China; b School of Nursing, Southern Medical University, Guangzhou, Guangdong, China; c Department of Pediatrics, Foshan Women and Children Hospital, Foshan, Guangdong, China; d School of Epidemiology and Public Health, Faculty of Medicine, University of Ottawa, Ottawa, ON, Canada; e Ottawa Hospital Research Institute, Department of Obstetrics & Gynecology, Faculty of Medicine University of Ottawa, Ottawa, ON, Canada; f Risk Science International, Ottawa, ON, Canada; g Department of Gynecology, Foshan Women and Children Hospital, Foshan, Guangdong, China; h The Affiliated Foshan Women and Children Hospital, School of Nursing, Guangdong Medical University, Foshan, Guangdong, China

**Keywords:** Allergic rhinitis, Atopic sensitization, Probiotics supplementation, Gut microbiota, Meta-analysis

## Abstract

**Background:**

Early-life gut microbial colonization is crucial for immune system development, but the efficacy of probiotic interventions during pregnancy or infancy in preventing allergic rhinitis (AR) and atopic sensitization (AS) remains unclear. This systematic review synthesized evidence from randomized controlled trials (RCTs) assessing probiotics for AR and AS prevention.

**Methods:**

Seven English and Chinese databases were systematically searched up to February 13, 2025. Eligible studies were RCTs with clearly defined probiotic interventions and specified AR or AS outcomes. Risk of bias was assessed using the Cochrane tool. Odds ratios (ORs) with 95% confidence intervals (CIs) were pooled using fixed-effects models.

**Results:**

Fourteen RCTs involving 5886 children were included. Probiotics did not significantly reduce the odds of AR at any age (≤ 1 year: OR_p_ = 0.73, 95% CI = 0.46–1.15, *P* = 0.17; > 1 year: OR_p_ = 0.95, 95% CI = 0.83–1.09, *P* = 0.46). However, probiotics significantly reduced AS odds in children over 1 year (OR_p_ = 0.87, 95% CI = 0.76–0.99, *P* = 0.04). Subgroup analyses revealed greater reductions in AS odds for postnatal interventions initiated after 6 months of age (OR_p_ = 0.82, 95% CI = 0.68–0.99, *P* = 0.04) and for combined prenatal-postnatal supplementation (OR_p_ = 0.85, 95% CI = 0.74–0.98, *P* = 0.03). Probiotics were also associated with transient increases in the abundance of targeted gut microbial strains.

**Conclusion:**

Probiotics during pregnancy and/or infancy may reduce AS odds in children over 1 year and correlate with age-related changes in gut microbial composition but failed to reduce AR odds at any age.

## Introduction

Allergic rhinitis (AR) is an immunoglobulin E (IgE)-mediated inflammatory response to inhaled allergens, primarily driven by atopic sensitization (AS), which triggers the production of allergen-specific IgE antibodies.^1^^,^^2^ The global prevalence of AR, particularly in children, has been steadily rising, making it one of the most common chronic conditions in high-income countries, with rates reaching up to 50% in some regions.^3^ In children, physician-diagnosed AR increased from 8.39% (2012–2015) to 19.87% (2016–2022).^4^ The annual economic burden of AR in the European Union is estimated at €30–50 billion.^5^ Beyond financial costs, AR significantly impairs quality of life, with approximately 30% of individuals experiencing cognitive and memory problems, and another 30% suffering from anxiety and depression. It also reduces work productivity in 82% of adults and adversely affects academic performance in 92% of children.^6^, ^7^, ^8^ Due to the complex pathophysiology and long-term burden of AR, early-life nutritional interventions during pregnancy, lactation, and infancy have gained attention as potential primary prevention strategies.^9^ However, current evidence indicates that interventions such as maternal vitamin D supplementation, prenatal fish and seafood intake, and hydrolyzed protein formula feeding do not significantly reduce AR odds.^10^, ^11^, ^12^

Growing research on the role of gut microbiota in allergic disease development has positioned probiotics as a promising prevention approach. The increasing incidence of allergic conditions has been partly explained by the “hygiene hypothesis”, which suggests that reduced microbial exposure early in life may contribute to immune dysregulation.^13^ Disruptions in gut microbiota composition—caused by factors such as cesarean delivery, formula feeding, antibiotic use, or parental atopy—are associated with increased odds of AR.^14^ Probiotics may help restore microbial balance by promoting beneficial strains like *Bifidobacterium*, potentially modulating immune responses.^15^ Probiotics are defined as live microorganisms that, when administered in adequate amounts, confer health benefits to the host.^16^

Initially proposed for the prevention and treatment of allergic diseases^17^ and probiotics have since been investigated extensively for their potential role in early-life allergy prevention, including AR.^18^^,^^19^ While some randomized controlled trials (RCTs) show that probiotics may reduce the odds of AR^20^ or AS, others report no significant effects.^22^^,^^23^ These inconsistent findings highlight the need for a comprehensive synthesis of the available evidence. Notably, delayed gut microbiota maturation in the first year of life has been significantly associated with allergic conditions, including atopic dermatitis, asthma, food allergies, and AR.^24^ Therefore, we conducted a meta-analysis of RCTs to assess whether probiotic supplementation during pregnancy and/or infancy reduces the odds of AR and AS in children.

## Material and methods

This study adhered to the latest Preferred Reporting Items for Systematic Reviews and Meta-Analyses (PRISMA-2020) guidelines.^25^ The review protocol was prospectively registered in PROSPERO (CRD42025637722).

### Search strategy

Two independent reviewers (ZXX and HQL) systematically searched 4 English databases (PubMed, Embase, Web of Science, Cochrane Library), and 3 Chinese databases (China National Knowledge Infrastructure [CNKI], Wanfang, Weipu Database [VIP]) for relevant trials published from inception to February 13, 2025. The search focused on the effectiveness of pregnancy and/or infancy probiotic supplementation in preventing AR. A combination of Medical Subject Headings (MeSH) and free-text keywords related to probiotics, allergic rhinitis, and infants was used, along with Boolean operators to maximize sensitivity. Reference lists of relevant studies and reviews were also screened to identify additional studies.^25^ Full search strategies for each database are presented (Supplementary Table 1).

### Eligibility criteria

Studies were included if they met the following criteria: (1) Infants born in families with a history of allergic disease; (2) Probiotic supplementation during pregnancy or infancy; (3) Control groups receiving a placebo; (4) Reported outcomes included occurrence of AR or AS; and (5) RCTs published in English or Chinese. Studies were excluded if they were duplicate publications, non-human studies or full text unavailable.

### Study selection

All search results were imported into Zotero (George Mason University, Fairfax, USA) for duplicate removal. Then, 2 independent reviewers (ZXX and HQL) performed a two-step screening process. First, titles and abstracts were screened for relevance based on the inclusion criteria. Second, the full texts of potentially eligible studies were reviewed to determine final inclusion. Discrepancies were resolved through discussion. If consensus could not be reached, a third reviewer (RHX) was consulted.

### Data extraction and management

Data extraction was independently performed by the 2 reviewers (ZXX and HQL) using a standardized and pre-tested Excel-based data extraction form (Microsoft Corporation, Redmond, USA). Extracted data included first author, year of publication, country of study, number of participants completing follow-up, probiotic intervention details (strain, timing, co-administration with prebiotics), use of placebo in control groups, participant age at final follow-up. The primary outcome was the incidence of AR. Secondary outcomes included the incidence of AS and data on gut microbiota composition. Any discrepancies were resolved by discussion, with input from a third reviewer (RHX) if necessary.

### Risk of bias assessment

Two reviewers (ZXX and HQL) independently assessed the risk of bias for each included RCT using the Cochrane Collaboration Risk of Bias tool.^26^ This tool evaluates randomization and allocation concealment, blinding of participants, personnel, and outcome assessors, completeness of outcome data, selective reporting, and other potential sources of bias. For trials with unclear methodology, corresponding authors were contacted for clarification. Disagreements were resolved through discussion or consultation with a third reviewer (RHX) as needed.

### Statistical analysis

Data were analyzed using RevMan 5.3 and Stata 14. Outcomes were expressed as odds ratios (ORs) with corresponding 95% confidence intervals (CIs). Heterogeneity across studies was assessed using the chi-square(Q) test and quantified with the *I*^*2*^ statistic. Substantial heterogeneity was defined as *P* < 0.10 for the Q-test or *I*^*2*^ > 50%. A fixed-effects Mantel–Haenszel model was employed for pooled effect estimation when heterogeneity was low, whereas a random-effects model was applied when substantial between-study heterogeneity was present. To explore potential sources of heterogeneity, subgroup analyses were conducted, and sensitivity analyses were performed by sequentially removing individual trials to assess their impact on the pooled results. Publication bias was assessed using funnel plots, Begg's test, and Egger's test. The trim-and-fill method was further applied to estimate the potential impact of unpublished studies on the pooled results. Statistical significance was set at *P* < 0.05.

## Results

### Literature search

**Fig. 1:**
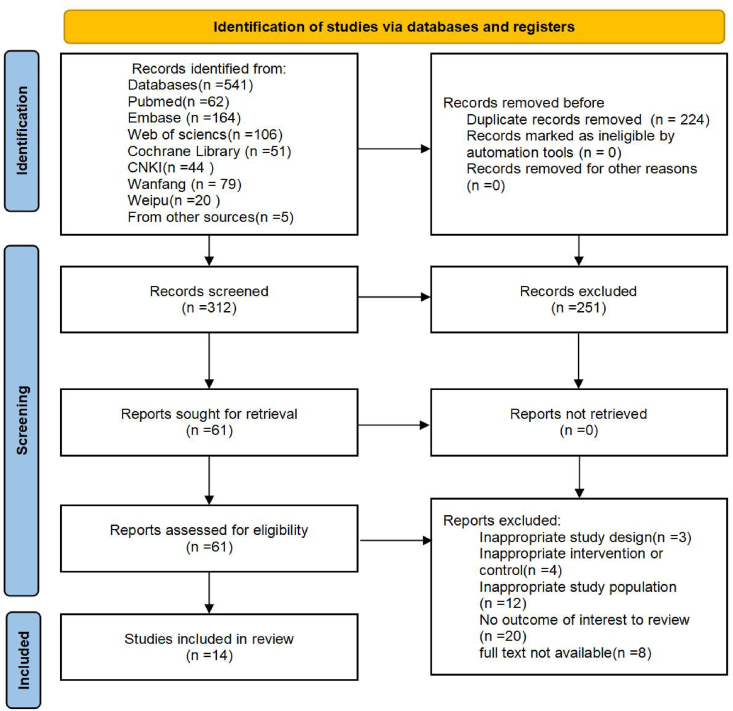
Flow chart of the search results.

A total of 536 potentially relevant studies were identified through electronic database searches and manual retrieval. The search yielded 62 records from PubMed, 164 from Embase, 51 from the Cochrane Library, 106 from Web of Science, 44 from CNKI, 79 from Wanfang, and 20 from VIP. Additionally, 5 records were obtained through manual searching. After removing 224 duplicate records in Zotero, 251 studies were excluded based on title and abstract screening. The remaining 61 records assessed in full text, of which 47 were excluded according to the predefined eligibility criteria. Ultimately, 14 studies were included in the meta-analysis. The study selection process is illustrated in Fig. 1.

### Study characteristics

Among the 14 included studies,^20^, ^21^, ^22^, ^23^^,^^27^, ^28^, ^29^, ^30^, ^31^, ^32^, ^33^, ^34^, ^35^, ^36^ 10 trials involved both pregnant mothers and their infants,^20^, ^21^, ^22^, ^23^^,^^28^^,^^30^, ^31^, ^32^, ^33^^,^^36^ while 4 focused solely on infants.^27^^,^^29^^,^^34^^,^^35^ All included studies encompassed a total of 5886 infants. Five trials investigated the effects of single probiotic strain,^27^, ^28^, ^29^^,^^32^ while 9 utilized multi-strains formulations.^20^^,^^22^^,^^27^^,^^30^^,^^31^^,^^33^, ^34^, ^35^, ^36^ Three of these trials administered synbiotic interventions (probiotic-prebiotic combinations).^22^^,^^31^^,^^36^ In all studies, infants were classified as ‘high risk’ if they had at least 1 first-degree relative with atopic eczema, AR or asthma.^37^ The characteristics of the included studies are summarized in Table 1. The outcome measures were evaluated through various approaches, including parental proxy reports, clinician-monitored follow-ups, symptom-focused structured interviews, physical examinations, skin prick tests (SPT), and allergen-specific IgE profiling. Our meta-analysis incorporated original trials with their follow-up studies. For example, both the initial trial by Abrahamsson et al. (2007)^23^ and its 6-year follow-up data (Abrahamsson et al., 2013)^28^ were included. In contrast, the primary trial by Kukkonen et al.^38^ was excluded due to incomplete data, but its 3 follow-up studies^22^^,^^31^^,^^36^ were included for their independent outcome reporting. Similarly, the primary trial by Wickens et al.^39^ was excluded due to incomplete datasets, while its 2 follow-up studies—the 4-year interim outcomes^20^ and 6-year final follow-up^21^—were included as they independently reported novel endpoint measures. Likewise, the primary trial by Niers et al.,^40^ was excluded, while its 6-year follow-up^33^ was incorporated.

**Table 1:** (Continued) Characteristics of 14 RCTs included in the meta-analysis.

Author, year	Country	Number of patients completed the follow-up (treated/control)	Probiotic treatment	Added prebiotic	Intervention -Start of treatment -End of treatment	Outcome(s) reported	Age at final evaluation (years old)
Zhang XX, 2011	China	75/75	*Lactobacillus*	No	S: from birth E: 6 months of age	AR	1.5
Ou CY, 2012	China	72/72	*Lactobacillus* GG	No	S: from gestational week 24 E: 6 months of age	AR; AS	3
Jensen MP, 2012	Australia	58/50	*Lactobacillus acidophilus*	No	S: from birth E: 6 months of age	AR; AS	5
Abrahamsson TR, 2007	Sweden	95/93	*L. reuteri*	No	S: from gestational week 36 E: 12 months of age	AR; AS	2
Abrahamsson TR, 2013	Sweden	94/90	*L. reuteri*	No	S: from gestational week 36 E: 12 months of age	AR; AS	7
Roβberg S, 2020	Germany	200/202	*Escherichia coli* *Enterococcus faecalis*	No	S: from 5 weeks before birth E: 31 weeks of age	AR; AS	11
Loo EX, 2014	Singapore	124/121	*Bifidobacterium longum* *Lactobacillus rhamnosus*	No	S: from birth E: 6 months of age	AR; AS	5
Soh SE, 2008	Singapore	124/121	*Bifidobacterium longum* *Lactobacillus rhamnosus*	No	S: from birth E: 6 months of age	AS	1
Kuitunen M, 2009	Finland	445/446	*Lactobacillus* GG *L rhamnosus* *Bifidobacterium breve* *Propionibacterium freudenreichii* ssp. *shermanii* JS	Yes	S: from 36 weeks of gestation E: 6 months of age	AR; AS; fecal samples	5

Author, year	Country	Number of patients completed the follow-up (treated/control)	Probiotic treatment	Added prebiotic	Intervention -Start of treatment -End of treatment	Outcome(s) reported	Age at final evaluation (years old)
Kallio S, 2019	Finland	213/246	*Lactobacillus* GG *L rhamnosus* *Bifidobacterium breve* *Propionibacterium freudenreichii* ssp. *shermanii* JS	Yes	S: from 36 weeks of gestation E: 6 months of age	AR; AS	13
Peldan P, 2017	Finland	407/400	*Lactobacillus* GG *L rhamnosus* *Bifidobacterium breve* *Propionibacterium freudenreichii* ssp. *shermanii* JS	Yes	S: from 36 weeks of gestation E: 6 months of age	AR	10
Wickens K, 2012	New Zealand	146/143	*L rhamnosus* HN001 *B animalis subsp lactis* HN019	No	S: from 35 weeks of gestation E: 24 months of age	AR; AS; fecal samples	4
Wickens K, 2013	New Zealand	144/144	*L rhamnosus* HN001 *B animalis subsp lactis* HN019	No	S: from 35 weeks of gestation E: 24 months of age	AR; AS	6
Gorissen DM, 2014	Netherlands	39/44	*Bifidobacterium bifidum* *Bifidobacterium lactis* *Lactococcus lactis*	No	S: from 36 weeks before birth E: 12 months of age	AR	6

Abbreviations: AR, allergic rhinitis; AS, atopic sensitization

### Risk of bias assessment

**Fig. 2:**
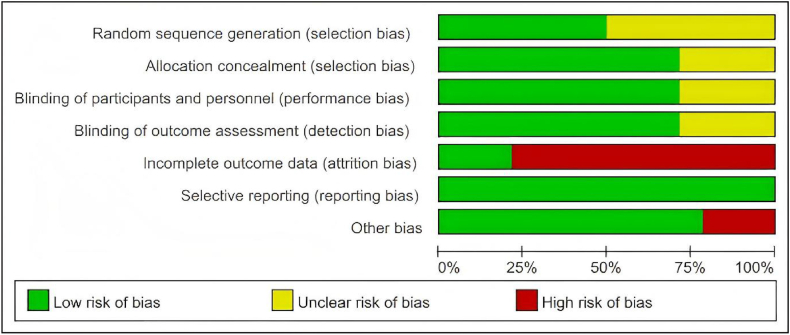
Summary of bias risk of included studies

The risk of bias summary is shown in Fig. 2. Of the 14 trials included, 7 reported adequate methods of randomization,^20^, ^21^, ^22^, ^23^^,^^28^^,^^34^^,^^35^ most trials employed blinding of outcome assessment (n = 10) and blinding of participants and personnel (n = 10).^22^^,^^23^^,^^27^^,^^28^^,^^31^, ^32^, ^33^, ^34^, ^35^, ^36^ However, 11 trials were assessed as having high risk of attrition bias due to substantial loss to follow-up and resulting incomplete outcome data.^20^, ^21^, ^22^^,^^27^^,^^28^^,^^30^^,^^31^^,^^33^, ^34^, ^35^, ^36^

### Probiotics and AR

#### Infant aged ≤ 1 year

**Fig. 3:**
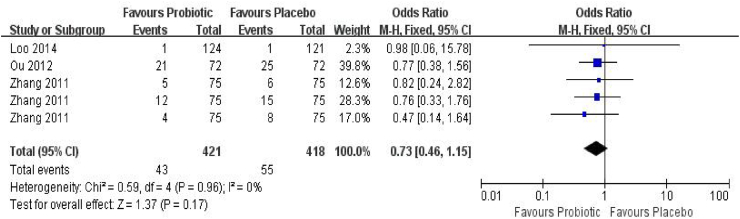
Forest plot of the association between probiotic supplementation and AR in infants ≤1 year of age. M–H: Mantel–Haenszel method.

Three RCTs,^29^^,^^32^^,^^35^ involving 839 children (421 in the probiotic group and 418 in the control group), evaluated AR incidence in infants aged ≤1 year (Fig. 3). The incidence of AR was 10.2% in the probiotic group and 13.2% in the placebo group, yielding no statistically significant difference (OR_p_ = 0.73, 95% CI = 0.46–1.15, *P* = 0.17). Heterogeneity was negligible (*I*^*2*^ = 0%, *P* = 0.96), supporting the use of a fixed-effects model. No evidence of publication bias was detected (Begg's test *P* = 0.99; Egger's test *P* = 0.89). Trim-and-fill analysis indicated no missing studies, and the pooled estimate remained unchanged (OR_p_ = 0.73, 95% CI = 0.47–1.15) (Supplementary Figure 1). Notably, Zhang et al.^29^ reported AR outcomes at 3, 6, and 12 months of age.

#### Children aged > 1 year

**Fig. 4:**
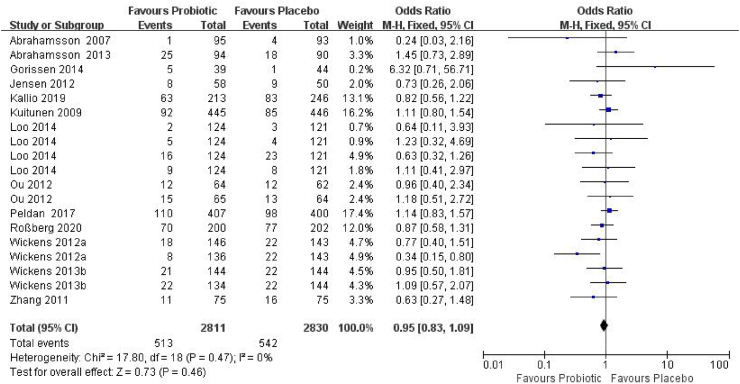
Forest plot of the association between probiotic supplementation and AR in children >1 year of age. M–H: Mantel–Haenszel method

Thirteen RCTs,^20^, ^21^, ^22^, ^23^^,^^27^, ^28^, ^29^, ^30^, ^31^, ^32^, ^33^^,^^35^^,^^36^ involving 5641 children (2811 in the probiotic group and 2830 in the control group) were included (Fig. 4). The incidence of AR was 18.2% in the probiotic group and 19.2% in the placebo group (OR_p_ = 0.95, 95% CI = 0.83–1.09, *P* = 0.46). A fixed-effects model was applied due to low heterogeneity (*I*^*2*^ = 0%, *P* = 0.47). No publication bias was detected (Begg's test *P* = 0.42; Egger's test *P* = 0.38). Trim-and-fill analysis imputed no studies, and the pooled estimate remained unchanged (OR_p_ = 0.95, 95% CI = 0.83∼1.09) (Supplementary Figure 2). As shown in Fig. 4, the study by Loo et al.^35^ contributed outcomes at 2, 3, 4, and 5 years of age, while Ou et al.^32^ provided outcomes at 1.5 and 3 years. Wickens et al.^20^ reported 4-year outcomes for both *Lactobacillus rhamnosus* HN001 and *Bifidobacterium animalis* subsp *lactis* strain HN019 compared to a placebo group, and Wickens et al.^21^ extended the follow-up to 6-year for both probiotic strains within the same cohort.

### Probiotics and AS

#### Infant aged **≤** 1 year

**Fig. 5:**
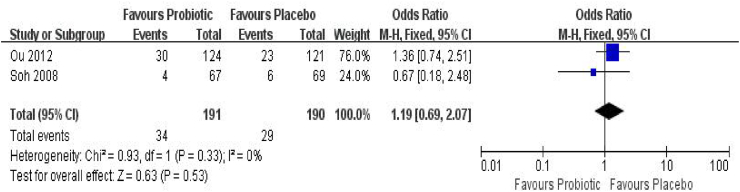
Forest plot of the association between probiotic supplementation and AS at≤1 year of age. M–H: Mantel–Haenszel method

Two trials^32^^,^^34^ including 381 infants contributed data on the incidence of AS in infants aged **≤** 1 year for the meta-analysis (Fig. 5). The incidence of AS was 17.8% in the probiotics group and 15.3% in the placebo group (OR_p_ = 1.19, 95%CI = 0.69–2.07, *P* = 0.53, *I*^*2*^ = 0%). Begg's test indicated no publication bias (*P* = 0.32), and Egger's test was not conducted due to the small number of studies. Trim-and-fill analysis imputed no studies, and the pooled estimate remained unchanged (OR_p_ = 1.20, 95% CI = 0.69–2.09) (Supplementary Figure 3). As this pooled estimate is based on only 2 studies, it should be interpreted with caution due to the limited statistical power.

#### Children aged > 1 year

**Fig. 6:**
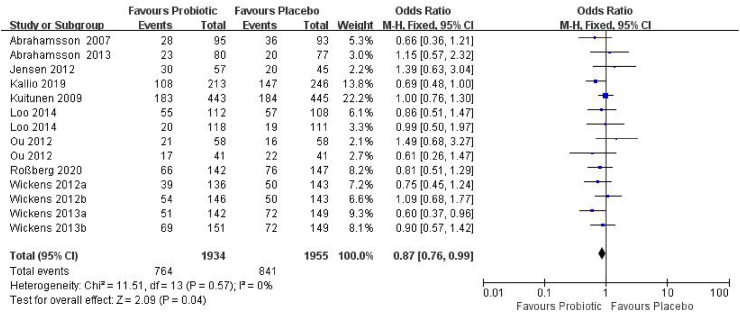
Forest plot of the association between probiotic supplementation and AS at > 1 year of age. M–H: Mantel–Haenszel method

This analysis included 10 RCTs,^20^, ^21^, ^22^, ^23^^,^^27^^,^^28^^,^^30^, ^31^, ^32^^,^^35^ involving 3889 children (1934 in the probiotic group and 1955 in the control group) (Fig. 6). The results revealed that the incidence of AS was lower in the probiotic group compared to the control group (39.5% vs 43.0%), representing a statistically significant reduction in odds (OR_p_ = 0.87, 95% CI = 0.76–0.99, *P* = 0.04). Heterogeneity was minimal (*I*^*2*^ = 0%, *P* = 0.57), supporting the use of a fixed-effects model.

**Fig. 7:**
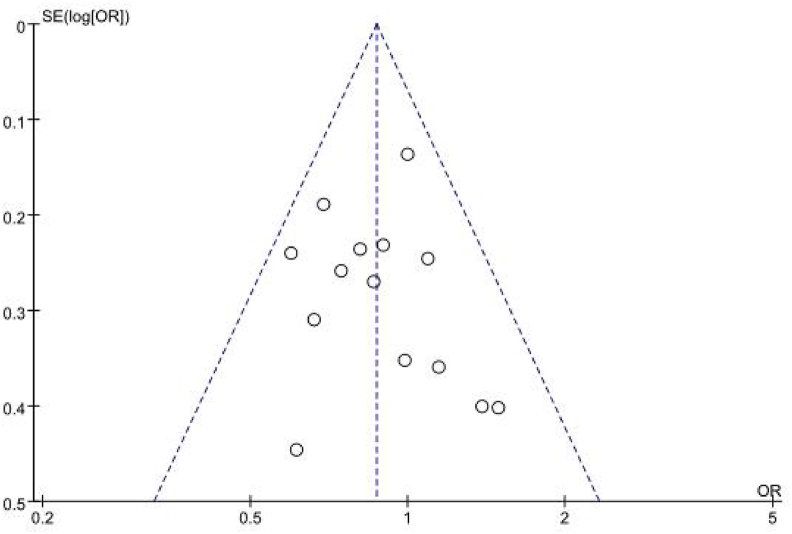
Funnel plot of trials on probiotic supplementation and prevention of AS at > 1 year of age with pseudo 95% confidence limits

In the forest plot, the diamond representing the pooled effect size was located to the left of the line of null effect and did not cross it, providing that early probiotic supplementation was effective in reducing the odds of AS in children aged > 1 year (Fig. 7). No evidence of publication bias was detected (Begg's test *P* = 0.30; Egger's test *P* = 0.78). Trim-and-fill analysis suggested no missing studies, and the pooled estimate remained unchanged (OR_p_ = 0.87, 95% CI = 0.76∼0.99) (Supplementary Figure 4).

### Subgroup analyses

No significant subgroup differences were observed in the effect of probiotics on reducing the odds of AR across probiotic formulations (single strain: OR_p_ = 0.95; multi-strain: OR_p_ = 0.86; *P* = 0.53), randomization quality (low risk: OR_p_ = 0.93; high/unclear risk: OR_p_ = 0.91; *P* = 0.83), attrition bias (low risk: OR_p_ = 0.97; high/unclear risk: OR_p_ = 0.67; *P* = 0.09), or geographic regions (Asia: OR_p_ = 0.80; Europe: OR_p_ = 1.03; Oceania: OR_p_ = 0.77; *P* = 0.14) (Supplementary Figure 5). Similarly, no significant subgroup differences were found in the effect of probiotics on reducing the odds of AS across probiotic formulations (single strain: OR_p_ = 0.95; multi-strain: OR_p_ = 0.87; *P* = 0.65), randomization quality (low risk: OR_p_ = 0.91; high/unclear risk: OR_p_ = 0.77; *P* = 0.27), attrition bias (low risk: OR_p_ = 0.90; high/unclear risk: OR_p_ = 0.66; *P* = 0.29), or geographic regions (Asia: OR_p_ = 1.00; Europe: OR_p_ = 0.86; Oceania: ORp = 0.85; *P* = 0.64) (Supplementary Figure 6). In addition, subgroup analyses revealed varying effectiveness of probiotic interventions on AS in children over 1 year of age under different conditions. Prolonged probiotic administration during infancy (ie, duration > 6 months) was associated with a significant protective effect (OR_p_ = 0.82; 95% CI = 0.68–0.99; *P* = 0.04), while shorter interventions (≤ 6 months) showed no statistically significant benefit (OR = 0.92; *P* = 0.34). No significant heterogeneity was observed between these subgroups (*P* = 0.93). Maternal-infant combined interventions significantly reduced AS odds (OR_p_ = 0.85, *P* = 0.03), whereas infant-only interventions showed no significant effect (OR_p_ = 1.00, *P* = 0.99). Age-stratified analysis indicated a stronger protective trend during adolescence (OR_p_ = 0.93, *P* = 0.08), though early and late childhood follow-ups showed non-significant outcomes, with no significant heterogeneity across age groups (*P* = 0.67). The addition of prebiotics did not significantly modify the intervention efficacy (prebiotic-supplemented group OR_p_ = 0.88 vs non-supplemented group OR_p_ = 0.86, between-group *P* = 0.89) (Supplementary Table 2).

### Probiotics and gut microbiota

Two studies^20^^,^^22^ examined the effects of probiotic supplementation during pregnancy and infancy on gut microbiota composition. Wickens et al^20^ demonstrated that although probiotic supplementation temporarily increased fecal levels of administered strains, it did not support sustained colonization of the distal gut. Fecal DNA analysis showed that only 8.5% of children had detectable levels of *B. animalis subsp. lactis* HN019 at 4 years after discontinuing supplementation, whereas *Lactobacillus rhamnosus* HN001 was detectable in 33%. Detection rates for HN001 were lower in both probiotic groups compared with the placebo group, but these differences were not statistically significant.

Consistent with this transient colonization pattern, Kukkonen et al^38^ reported that at 6 months of age, colonization rates of *L. rhamnosus GG*, *L. rhamnosus* LC705, and *Propionibacterium JS* in the probiotic group were markedly higher than in the placebo group (eg, *L. rhamnosus GG*: 91.3% vs 23.1%, *P* < 001), whereas by 24 months these differences were no longer significant (eg, 41.5% vs 55.3%, *P* = 0.195). Kuitunen et al^22^ demonstrated delayed recovery of *bifidobacteria* in cesarean-delivered infants receiving a placebo, with fecal prevalence reaching only 57% at 6 months. This deficit was fully corrected through combined probiotic and prebiotic supplementation, achieving 100% bifidobacteria colonization (*P* = 0.036). In contrast, vaginally delivered infants showed no significant differences in *Bifidobacteria* colonization between treatment arms.

## Discussion

### Main findings

Proactive approaches are essential to reducing the global burden of AR. This meta-analysis of 14 RCTs involving 5886 children demonstrated that probiotic supplementation during pregnancy or infancy did not significantly lower the incidence of AR at any age. However, probiotics showed clinically meaningful protective effects against AS in children over 1 year of age. Notably, interventions combining prenatal and postnatal supplementation or extended beyond 6 months of age were associated with significant reductions in AS odds. In contrast, interventions limited to infancy or shorter than 6 months showed no benefit, underscoring the critical role of timing and duration in designing effective probiotic-based allergy prevention strategies.

### Effectiveness of probiotics in AR prevention

Our findings align with those of Luo et al,^41^ who reported no significant preventive effect of probiotics on AR in their analysis of 14 trials involving 3501 infants. By incorporating a larger body of evidence from studies with longer follow-up periods, our meta-analysis enhances the robustness and generalizability of the conclusions. Several methodological and etiological challenges may explain the absence of significant findings. First, diagnostic misclassification poses a major challenge. Many included trials relied on parent-reported ISAAC questionnaires,^20^^,^^22^^,^^30^^,^^31^^,^^36^ wherein symptoms such as nasal itching and sneezing in infants are often indistinguishable from common respiratory infections. This reliance on subjective reports introduces substantial recall bias and likely underestimate the AR incidence. Second, incomplete outcome data severely limit the validity of the findings. Only 3 trials^23^^,^^29^^,^^32^ reported complete datasets, while others suffered from significant attrition. For example, Kallio et al^31^ reported an attrition rate as high as 53%. Such losses to follow-up limit interpretability, particularly for long-term outcomes. Future studies should prioritize AR as a primary endpoint, using standardized ARIA diagnostic criteria and incorporating objective biomarkers such as nasal eosinophil counts or serum-specific IgE.^42^^,^^43^ Additionally, longitudinal designs with developmentally appropriate, frequent follow-ups and pediatric-friendly retention strategies are needed to minimize attrition and recall bias.

### Effectiveness of probiotics in AS prevention

This meta-analysis demonstrated that combined prenatal and postnatal probiotic interventions significantly reduced the odds of AS in children (OR_p_ = 0.85, *P* = 0.03), consistent with the findings of Elazab et al.^44^ Although Elazab et al^44^ included 14 trials primarily involving low-risk populations without a family history of atopy, our study focused on high-risk children (ie, with at least 1 allergic parent). Despite these differences, both analyses found that combined prenatal-postnatal probiotic supplementation conferred a significant protective effect against AS (*P* = 0.035), whereas postnatal-only interventions did not (*P* = 0.825).

This consistency underscores the pivotal role of the maternal-fetal immune-microbial axis in probiotic-mediated allergy prevention. Rautava et al^45^ have shown that maternal gut microbiota can influence fetal immune development through placental metabolites and immune cells signaling, thereby modulating the neonatal Th1/Th2 balance and shaping early gut colonization. Postnatal probiotic supplementation may reinforce this immunological programming, helping to sustain a balanced immune response and reduce early-life Th2 dominance, which is implicated in the development of AS. Interestingly, although age-stratified subgroup analyses did not identify statistically significant effects of probiotics within individual age groups, the pooled analysis revealed a protective effect of combined prenatal-postnatal interventions. This apparent discrepancy may reflect limitations in statistical power within smaller subgroups, particularly in older children (> 6 years subgroup, which included only 3 trials). In contrast, pooled analyses aggregate data across studies, enhancing sensitivity to detect modest but clinically relevant effects.

### Probiotic supplementation and gut microbiota

Wickens et al^20^ found that probiotic supplementation during pregnancy and infancy transiently increased the abundance of targeted strains (eg, specific *Bifidobacterium* or *Lactobacillus* species) in the infant gut. Our findings align with the hygiene hypothesis, which suggests that early life is a critical window for gut microbial colonization and immune system programming.^46^, ^47^, ^48^ During this period, infants are exposed to a diverse array of microorganisms, some of which become stably integrated into in the gut mircorbiome.^49^ Notably, combined probiotic-prebiotic interventions fully restored *Bifidobacterium* colonization in cesarean-delivered infants (100% vs 57%, *P* = 0.036),^22^ likely compensating for microbiota deficiencies associated with cesarean birth.

### Strengths and limitations

This meta-analysis has several strengths. First, by focusing exclusively on high-risk pediatric populations, we significantly reduced inter-study heterogeneity, as evidenced by the negligible statistical heterogeneity (*I*^*2*^ = 0%) across primary outcomes. Second, the inclusion of 9 follow-up studies with extended observation periods (up to 13 years) enabled robust evaluation of the sustained probiotics effects. Moreover, our dual focus on clinical outcomes (AR/AS prevention) and mechanistic insights (gut microbiota modulation) offers a more comprehensive perspective than previous reviews primarily centered on symptom.

However, several limitations should be considered when interpreting these findings. Some subgroup analyses were based on very few studies (eg, only 2 for AS in infants ≤ 1 year), limiting statistical power. Follow-up time points varied across trials, complicating age-specific outcome assessment. In addition, standardized microbiota data were available from only 2 studies, precluding pooled analysis and highlighting the need for future trials with consistent follow-ups and integrated multi-omics approaches.

## Conclusion

This review suggests that probiotic supplementation during pregnancy or infancy is associated with reduced odds of AS in children over 1 year and correlates with age-related changes in gut microbial composition. However, probiotics fail to reduce the odds of AR at any age.

## Author contributions

Zhengxiu Xiao and Ri-Hua Xie conceived the review. Zhengxiu Xiao and Haiqin Luo designed the search strategy and performed the searches. Zhengxiu Xiao, Haiqin Luo and Ri-Hua Xie performed quality assessment and data extraction. Zhengxiu Xiao performed the meta-analysis and drafted the manuscript. Hao Liu, Hui Chen, Daniel Krewski, Jun Bai, Shi Wu Wen, Wang Liao, Huiling Shang, and Ri-Hua Xie provided critical feedback and edits to the draft. Ri-Hua Xie approved the final version of the manuscript to be published. All authors provided final approval of the manuscript and agreed to be accountable for all aspects of the work.

## Ethics statement

Not applicable.

## Submission declaration

Not applicable.

## Declaration of Generative AI and AI-assisted technologies in the writing process

The authors declare that generative artificial intelligence (AI) and AI-assisted technologies were not employed in the conception of the study, the development of its methodology, the analysis and interpretation of data, or the formulation of scientific, clinical, pedagogical, or medical conclusions. An AI-assisted tool (ChatGPT) was used only for correcting English spelling and grammar.

## Funding

This systematic review was in part funded by the Guangdong Basic and Applied Basic Research Foundation (#2024A1515010343), the Foshan Competitive Talent Support Project (#2024013), and the Top Talent Program of Foshan Women & Children Hospital (#FY2022011).

## Declaration of competing interest

None declared.

## References

[1] Shade K.T.C., Conroy M.E., Washburn N. (2020). Sialylation of immunoglobulin E is a determinant of allergic pathogenicity. Nature.

[2] Bousquet J., Anto J.M., Bachert C. (2020). Allergic rhinitis. Nat Rev Dis Primer.

[3] Bousquet P.J., Leynaert B., Neukirch F. (2008). Geographical distribution of atopic rhinitis in the european community respiratory health survey I. Allergy.

[4] Licari A., Magri P., De Silvestri A. (2023). Epidemiology of allergic rhinitis in children: a systematic review and meta-analysis. J Allergy Clin Immunol Pract.

[5] Zuberbier T., Lötvall J., Simoens S., Subramanian S.V., Church M.K. (2014). Economic burden of inadequate management of allergic diseases in the european union: a GA(2) LEN review. Allergy.

[6] Meltzer E.O., Blaiss M.S., Naclerio R.M. (2012). Burden of allergic rhinitis: allergies in America, Latin America, and Asia-Pacific adult surveys. Allergy Asthma Proc.

[7] Meltzer E.O., Farrar J.R., Sennett C. (2017). Findings from an online survey assessing the burden and management of seasonal allergic rhinoconjunctivitis in US patients. J Allergy Clin Immunol Pract.

[8] Blaiss M.S., Hammerby E., Robinson S., Kennedy-Martin T., Buchs S. (2018). The burden of allergic rhinitis and allergic rhinoconjunctivitis on adolescents: a literature review. Ann Allergy Asthma Immunol Off Publ Am Coll Allergy Asthma Immunol.

[9] Arshad H., Lack G., Durham S.R., Penagos M., Larenas-Linnemann D., Halken S. (2024). Prevention is better than cure: impact of allergen immunotherapy on the progression of airway disease. J Allergy Clin Immunol Pract.

[10] Osborn D.A., Sinn J.K., Jones L.J. (2018). Infant formulas containing hydrolysed protein for prevention of allergic disease. Cochrane Database Syst Rev.

[11] Yepes-Nuñez J.J., Brożek J.L., Fiocchi A. (2018). Vitamin D supplementation in primary allergy prevention: systematic review of randomized and non-randomized studies. Allergy.

[12] Stratakis N., Roumeliotaki T., Oken E. (2017). Fish and seafood consumption during pregnancy and the risk of asthma and allergic rhinitis in childhood: a pooled analysis of 18 European and US birth cohorts. Int J Epidemiol.

[13] Shreiner A., Huffnagle G.B., Noverr M.C. (2008). The “microflora hypothesis” of allergic disease. Adv Exp Med Biol.

[14] Donald K., Finlay B.B. (2023). Early-life interactions between the microbiota and immune system: impact on immune system development and atopic disease. Nat Rev Immunol.

[15] Kallio S., Jian C., Korpela K. (2024). Early-life gut microbiota associates with allergic rhinitis during 13-year follow-up in a Finnish probiotic intervention cohort. Microbiol Spectr.

[16] Hill C., Guarner F., Reid G. (2014). Expert consensus document. The international scientific association for probiotics and prebiotics consensus statement on the scope and appropriate use of the term probiotic. Nat Rev Gastroenterol Hepatol.

[17] Ismail I.H., Licciardi P.V., Tang M.L. (2013). Probiotic effects in allergic disease. J Paediatr Child Health.

[18] Wickens K., Barthow C., Mitchell E.A. (2018). Effects of Lactobacillus rhamnosus HN001 in early life on the cumulative prevalence of allergic disease to 11 years. Pediatr Allergy Immunol Off Publ Eur Soc Pediatr Allergy Immunol.

[19] Súkeníková L., Černý V., Thon T. (2023). Effect of early postnatal supplementation of newborns with probiotic strain E. coli O83:K24:H31 on allergy incidence, dendritic cells, and microbiota. Front Immunol.

[20] Wickens K., Black P., Stanley T.V. (2012). A protective effect of Lactobacillus rhamnosus HN001 against eczema in the first 2 years of life persists to age 4 years. Clin Exp Allergy J Br Soc Allergy Clin Immunol.

[21] Wickens K., Stanley T.V., Mitchell E.A. (2013). Early supplementation with Lactobacillus rhamnosus HN001 reduces eczema prevalence to 6 years: does it also reduce atopic sensitization?. Clin Exp Allergy J Br Soc Allergy Clin Immunol.

[22] Kuitunen M., Kukkonen K., Juntunen-Backman K. (2009). Probiotics prevent IgE-associated allergy until age 5 years in cesarean-delivered children but not in the total cohort. J Allergy Clin Immunol.

[23] Abrahamsson T.R., Jakobsson T., Böttcher M.F. (2007). Probiotics in prevention of IgE-associated eczema: a double-blind, randomized, placebo-controlled trial. J Allergy Clin Immunol.

[24] Hoskinson C., Dai D.L.Y., Del Bel K.L. (2023). Delayed gut microbiota maturation in the first year of life is a hallmark of pediatric allergic disease. Nat Commun.

[25] Page M.J., McKenzie J.E., Bossuyt P.M. (2021). The PRISMA 2020 statement: an updated guideline for reporting systematic reviews. BMJ.

[26] Cumpston M., Li T., Page M.J. (2019). Updated guidance for trusted systematic reviews: a new edition of the cochrane handbook for systematic reviews of interventions. Cochrane Database Syst Rev.

[27] Jensen M.P., Meldrum S., Taylor A.L., Dunstan J.A., Prescott S.L. (2012). Early probiotic supplementation for allergy prevention: long-term outcomes. J Allergy Clin Immunol.

[28] Abrahamsson T.R., Jakobsson T., Björkstén B., Oldaeus G., Jenmalm M.C. (2013). No effect of probiotics on respiratory allergies: a seven-year follow-up of a randomized controlled trial in infancy. Pediatr Allergy Immunol Off Publ Eur Soc Pediatr Allergy Immunol.

[29] Zhang xiuxia PJ. (2011). Observation of clinical effect of orally lactobacillus in early stage on infant allergic disease. Anhui Med J.

[30] Roßberg S., Keller T., Icke K. (2020). Orally applied bacterial lysate in infants at risk for atopy does not prevent atopic dermatitis, allergic rhinitis, asthma or allergic sensitization at school age: follow-up of a randomized trial. Allergy.

[31] Kallio S., Kukkonen A.K., Savilahti E., Kuitunen M. (2019). Perinatal probiotic intervention prevented allergic disease in a caesarean-delivered subgroup at 13-year follow-up. Clin Exp Allergy J Br Soc Allergy Clin Immunol.

[32] Ou C.Y., Kuo H.C., Wang L. (2012). Prenatal and postnatal probiotics reduces maternal but not childhood allergic diseases: a randomized, double-blind, placebo-controlled trial. Clin Exp Allergy J Br Soc Allergy Clin Immunol.

[33] Gorissen D.M.W., Rutten N.B.M.M., Oostermeijer C.M.J. (2014). Preventive effects of selected probiotic strains on the development of asthma and allergic rhinitis in childhood. The panda study. Clin Exp Allergy J Br Soc Allergy Clin Immunol.

[34] Soh S.E., Aw M., Gerez I. (2009). Probiotic supplementation in the first 6 months of life in at risk Asian infants--effects on eczema and atopic sensitization at the age of 1 year. Clin Exp Allergy J Br Soc Allergy Clin Immunol.

[35] Loo E.X.L., Llanora G.V., Lu Q., Aw M.M., Lee B.W., Shek L.P. (2014). Supplementation with probiotics in the first 6 months of life did not protect against eczema and allergy in at-risk Asian infants: a 5-year follow-up. Int Arch Allergy Immunol.

[36] Peldan P., Kukkonen A.K., Savilahti E., Kuitunen M. (2017). Perinatal probiotics decreased eczema up to 10 years of age, but at 5-10 years, allergic rhino-conjunctivitis was increased. Clin Exp Allergy J Br Soc Allergy Clin Immunol.

[37] Ní Chaoimh C., Lad D., Nico C. (2023). Early initiation of short-term emollient use for the prevention of atopic dermatitis in high-risk infants-the STOP-AD randomised controlled trial. Allergy.

[38] Kukkonen K., Savilahti E., Haahtela T. (2007). Probiotics and prebiotic galacto-oligosaccharides in the prevention of allergic diseases: a randomized, double-blind, placebo-controlled trial. J Allergy Clin Immunol.

[39] Wickens K., Black P.N., Stanley T.V. (2008). A differential effect of 2 probiotics in the prevention of eczema and atopy: a double-blind, randomized, placebo-controlled trial. J Allergy Clin Immunol.

[40] Niers L., Martín R., Rijkers G. (2009). The effects of selected probiotic strains on the development of eczema (the PandA study). Allergy.

[41] Luo X., Wang H., Liu H. (2024). Effects of probiotics on the prevention and treatment of children with allergic rhinitis: a meta-analysis of randomized controlled trials. Front Pediatr.

[42] Allergic Rhinitis and its Impact on Asthma (ARIA) Phase 4 (2018): Change Management in Allergic Rhinitis and Asthma Multimorbidity Using Mobile Technology - PubMed. Accessed March 25, 2025. https://pubmed.ncbi.nlm.nih.gov/30273709/. 10.1016/j.jaci.2018.08.049 30273709

[43] Brożek J.L., Bousquet J., Agache I. (2017). Allergic rhinitis and its impact on asthma (ARIA) guidelines-2016 revision. J Allergy Clin Immunol.

[44] Elazab N., Mendy A., Gasana J., Vieira E.R., Quizon A., Forno E. (2013). Probiotic administration in early life, atopy, and asthma: a meta-analysis of clinical trials. Pediatrics.

[45] Rautava S., Luoto R., Salminen S., Isolauri E. (2012). Microbial contact during pregnancy, intestinal colonization and human disease. Nat Rev Gastroenterol Hepatol.

[46] Mr P., Dp S. (2022). The hygiene hypothesis for allergy - conception and evolution. Front Allergy.

[47] Ray K. (2016). Gut microbiota: first steps in the infant gut microbiota. Nat Rev Gastroenterol Hepatol.

[48] Bokulich N.A., Chung J., Battaglia T. (2016). Antibiotics, birth mode, and diet shape microbiome maturation during early life. Sci Transl Med.

[49] Rey-Mariño A., Francino M.P. (2022). Nutrition, gut microbiota, and allergy development in infants. Nutrients.

