# Supplementary material for: Probiotic supplementation during pregnancy or infancy for the prevention of allergic rhinitis in infants: A systematic review and meta-analysis of Randomized controlled trials
Source: World Allergy Organ J. 2025 Oct 4;18(10):101124. doi: 10.1016/j.waojou.2025.101124 (PMC12513199; doi:10.1016/j.waojou.2025.101124)
Supplement: Multimedia component 1 [file mmc1.pdf]

A

```
Begg's Test

  adj. Kendall's Score (P-Q) =       0
          Std. Dev. of Score =    4.08
           Number of Studies =       5
                          z  =    0.00
                    Pr > |z| =   1.000
                          z  =   -0.24 (continuity corrected)
                    Pr > |z| =   1.000 (continuity corrected)


Egger's test
```

Std_Eff	Coef.	Std. Err.	t	P>|t|	[95% Conf. Interval]	
slope	-.2724113	.2744923	-0.99	0.394	-1.145968	.6011457
bias	-.0823046	.5316945	-0.15	0.887	-1.774394	1.609785

B

```
Meta-analysis (exponential form)

       |  Pooled      95% CI         Asymptotic      No. of
Method |     Est   Lower   Upper  z_value  p_value   studies
-------+----------------------------------------------------
Fixed  |   0.732   0.466   1.151   -1.351    0.177      5
Random |   0.732   0.466   1.151   -1.351    0.177

Test for heterogeneity: Q=  0.585 on 4 degrees of freedom (p= 0.965)
Moment-based estimate of between studies variance =  0.000

. metatrim logor _selogES,eform funnel

Note: default data input format (theta, se_theta) assumed.

Meta-analysis

       |  Pooled      95% CI         Asymptotic      No. of
Method |     Est   Lower   Upper  z_value  p_value   studies
-------+----------------------------------------------------
Fixed  |  -0.312  -0.764   0.141   -1.351    0.177      5
Random |  -0.312  -0.764   0.141   -1.351    0.177

Test for heterogeneity: Q=  0.585 on 4 degrees of freedom (p= 0.965)
Moment-based estimate of between studies variance =  0.000
```

Supplementary Figure 1. Publication bias assessment (A) and trim-and-fill analysis (B) for the meta-analysis of probiotic supplementation and AR risk in infants ≤ 1 year.

A

```
Tests for Publication Bias

Begg's Test

  adj. Kendall's Score (P-Q) =     -23
          Std. Dev. of Score =   28.58
           Number of Studies =      19
                           z =   -0.80
                    Pr > |z| =   0.421
                           z =    0.77 (continuity corrected)
                    Pr > |z| =   0.441 (continuity corrected)

Egger's test
------------------------------------------------------------------------------
     Std_Eff |     Coef.   Std. Err.      t    P>|t|     [95% Conf. Interval]
-------------+----------------------------------------------------------------
       slope |  .0697984    .1491178    0.47   0.646    -.2448126    .3844094
        bias | -.4360094     .481605   -0.91   0.378    -1.452107    .5800884
------------------------------------------------------------------------------
```

B

```
Meta-analysis

       |  Pooled      95% CI         Asymptotic      No. of
Method |     Est   Lower   Upper  z_value  p_value   studies
-------+----------------------------------------------------
Fixed  |  -0.049  -0.188   0.090   -0.689    0.491      15
Random |  -0.049  -0.188   0.090   -0.689    0.491

Test for heterogeneity: Q= 17.798 on 18 degrees of freedom (p= 0.469)
Moment-based estimate of between studies variance =  0.000

Trimming estimator: Linear
Meta-analysis type: Fixed-effects model

iteration |  estimate    Tn    # to trim     diff
----------+--------------------------------------
    1     |   -0.049     83        0          190
    2     |   -0.049     83        0            0

Note: no trimming performed; data unchanged

Filled
Meta-analysis (exponential form)

       |  Pooled      95% CI         Asymptotic      No. of
Method |     Est   Lower   Upper  z_value  p_value   studies
-------+----------------------------------------------------
Fixed  |   0.952   0.828   1.095   -0.689    0.491      15
Random |   0.952   0.828   1.095   -0.689    0.491

Test for heterogeneity: Q= 17.798 on 18 degrees of freedom (p= 0.469)
Moment-based estimate of between studies variance =  0.000
```

Supplementary Figure 2： Publication bias assessment (A) and trim-and-fill analysis (B) for the meta-analysis of probiotic supplementation and AR risk in children > 1 year.

A

Begg's Test

adj. Kendall's Score (P-Q) =	-1
Std. Dev. of Score =	1.00
Number of Studies =	2
z =	-1.00
Pr > |z| =	0.317
z =	0.00 (continuity corrected)
Pr > |z| =	1.000 (continuity corrected)

Egger's test

Std_Eff	Coef.	Std. Err.	t	P>|t|	[95% Conf. Interval]	
slope	.9311096	.	.	.	.	.
bias	-1.995971	.	.	.	.	.

B

Meta-analysis (exponential form)

Method	Pooled Est	95% CI Lower	95% CI Upper	Asymptotic z_value	Asymptotic p_value	No. of studies
Fixed	1.197	0.687	2.085	0.635	0.525	2
Random	1.197	0.687	2.085	0.635	0.525	

Test for heterogeneity: Q= 0.931 on 1 degrees of freedom (p= 0.335)
Moment-based estimate of between studies variance = 0.000

. metatrim logor _selogES,eform funnel

Note: default data input format (theta, se_theta) assumed.

Meta-analysis

Method	Pooled Est	95% CI Lower	95% CI Upper	Asymptotic z_value	Asymptotic p_value	No. of studies
Fixed	0.180	-0.375	0.735	0.635	0.525	2
Random	0.180	-0.375	0.735	0.635	0.525	

Test for heterogeneity: Q= 0.931 on 1 degrees of freedom (p= 0.335)
Moment-based estimate of between studies variance = 0.000

Supplementary Figure 3: Publication bias assessment (A) and trim-and-fill analysis (B) for the meta-analysis of probiotic supplementation and AS risk in infants ≤ 1 year.

A

```
Begg's Test

  adj. Kendall's Score (P-Q) =      19
          Std. Dev. of Score =   18.27
          Number of Studies =      14
                          z =    1.04
                   Pr > |z| =   0.298
                          z =    0.99 (continuity corrected)
                   Pr > |z| =   0.324 (continuity corrected)


Egger's test
```

Std_Eff	Coef.	Std. Err.	t	P>|t|	[95% Conf.	Interval]
slope	-.1951567	.201126	-0.97	0.351	-.6333727	.2430592
bias	.2396054	.8063522	0.30	0.771	-1.517285	1.996496

B

```
Meta-analysis

       |  Pooled      95% CI         Asymptotic      No. of
Method |     Est    Lower    Upper  z_value  p_value   studies
-------+----------------------------------------------------
Fixed  |  -0.139   -0.269   -0.008   -2.079    0.038      14
Random |  -0.139   -0.269   -0.008   -2.079    0.038

Test for heterogeneity: Q= 11.506 on 13 degrees of freedom (p= 0.569)
Moment-based estimate of between studies variance =  0.000

Trimming estimator: Linear
Meta-analysis type: Fixed-effects model

iteration |  estimate     Tn    # to trim     diff
----------+--------------------------------------
    1     |    -0.139     55          0        105
    2     |    -0.139     55          0          0

Note: no trimming performed; data unchanged

Filled
Meta-analysis (exponential form)

       |  Pooled      95% CI         Asymptotic      No. of
Method |     Est    Lower    Upper  z_value  p_value   studies
-------+----------------------------------------------------
Fixed  |   0.871    0.764    0.992   -2.079    0.038      14
Random |   0.871    0.764    0.992   -2.079    0.038

Test for heterogeneity: Q= 11.506 on 13 degrees of freedom (p= 0.569)
Moment-based estimate of between studies variance =  0.000
```

Supplementary Figure 4: Publication bias assessment (A) and trim-and-fill analysis (B) for the meta-analysis of probiotic supplementation and AS risk in children > 1 year.

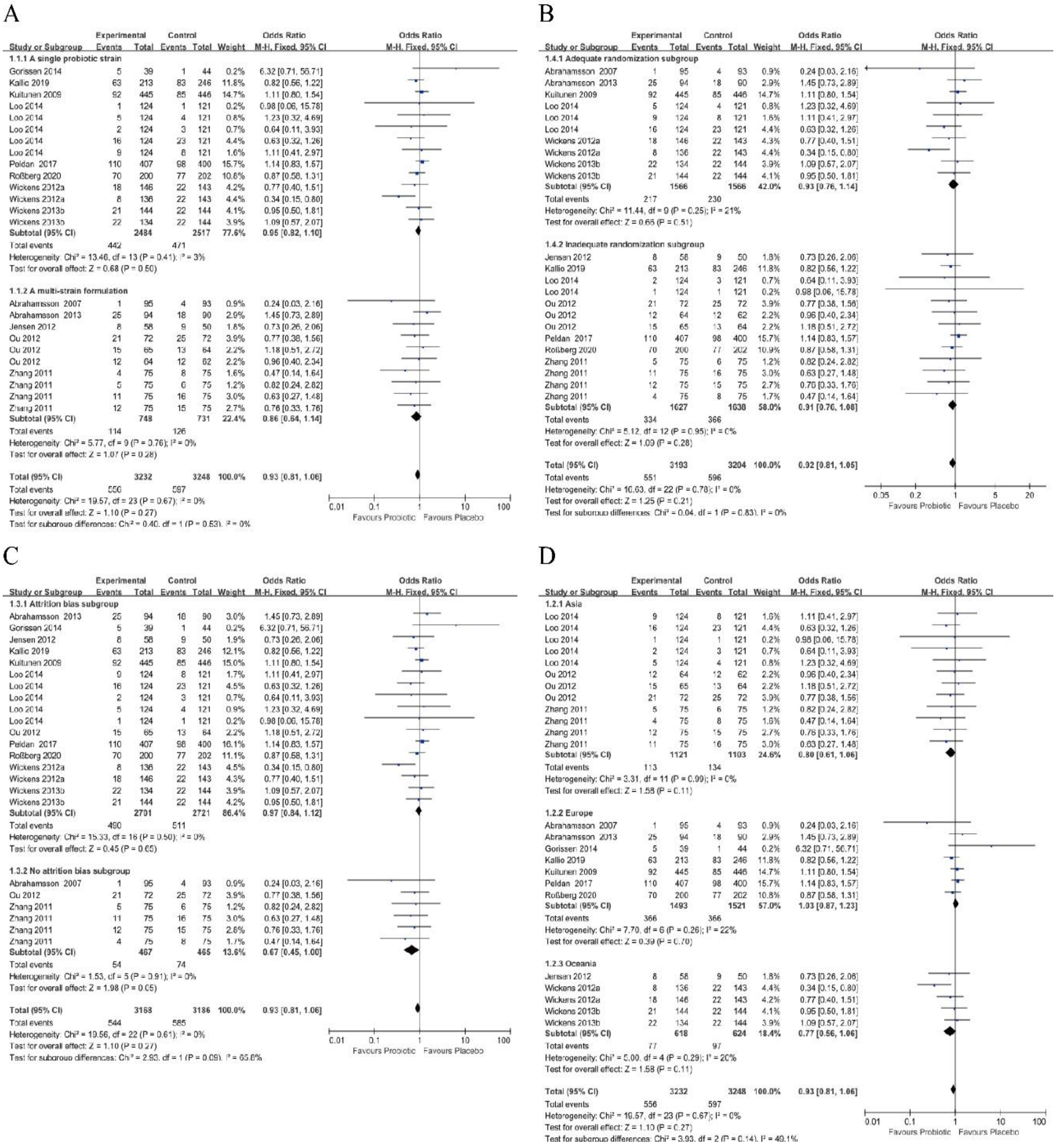

Supplementary Figure 5: Subgroup analyses of probiotics for AR prevention (A: formulation type; B: randomization adequacy; C: attrition bias; D: ethnicity).

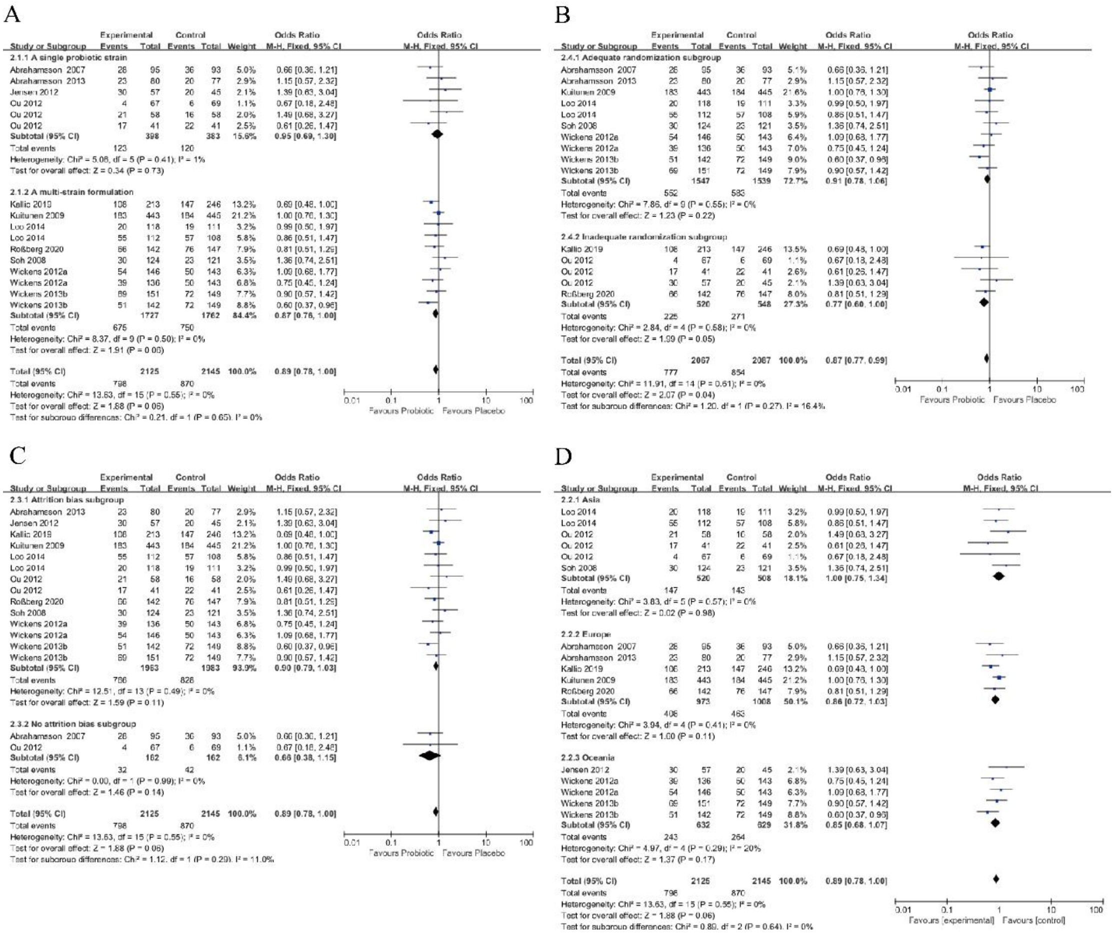

Supplementary Figure 6: Subgroup analyses of probiotics for AS prevention (A: formulation type; B: randomization adequacy; C: attrition bias; D: ethnicity).
