# Supplementary material for: Probiotic supplementation during pregnancy or infancy for the prevention of allergic rhinitis in infants: A systematic review and meta-analysis of Randomized controlled trials
Source: World Allergy Organ J. 2025 Oct 4;18(10):101124. doi: 10.1016/j.waojou.2025.101124 (PMC12513199; doi:10.1016/j.waojou.2025.101124)
Supplement: Multimedia component 2 [file mmc2.docx]

**Supplementary Table**

**Supplementary Table 1：**Search strategy.

| **Database** | **Search Terms** | **Search Field** | **Search Results** |
| --- | --- | --- | --- |
| PubMed | (((((("Infant"[Mesh]) OR (infant*)) OR (neonate*)) OR (newborn*)) OR (toddler*)) AND (("Probiotics"[Mesh]) OR (Probiotic*))) AND (("Rhinitis, Allergic"[Mesh]) OR (allergic rhinit*)) | All Fields | 62 |
| Embase | ('infant'/exp OR 'infant*' OR 'neonate*' OR 'newborn*' OR 'toddler*') AND ('probiotic'/exp OR 'probiotic' OR 'probiotics'/exp OR 'probiotics' OR 'probiotic agent'/exp OR 'probiotic agent') AND ('rhinitis allergica'/exp OR 'rhinitis allergica' OR 'rhinitis, allergic'/exp OR 'rhinitis, allergic' OR 'allergic rhinitis'/exp OR 'allergic rhinitis') | All Fields | 164 |
| WOS | #1:TS= (Infant OR Infant* OR Neonate* OR Newborn* OR toddler*)2,124,825  #2:TS= (Probiotics OR Probiotic*)96,190  #3:TS= ("Rhinitis, Allergic" OR "allergic rhinit*")51,371  #1AND #2AND #3 106 | Topic Search | 106 |
| Cochrane | #1 MeSH descriptor: [Infant] explode all trees 47086  #2 MeSH descriptor: [Probiotics] explode all trees 3862  #3 MeSH descriptor: [Rhinitis, Allergic] explode all trees 3895  #4 ((infant* OR neonate* OR newborn* OR toddler*)) (Word variations have been searched) 92925  #5 (Probiotic*) (Word variations have been searched) 11472  #6 ((rhinitis allergica) OR (rhinitis, allergic) OR (allergic rhinit*)) (Word variations have been searched) 9467  #7 #1 OR #4 92926  #8 #2 OR #5 11625  #9 #3 OR #6 9467  #10 # 7 AND #8 AND #9 51 | All Fields | 51 |
| VIP | (neonate OR newborn OR "early life" OR infant OR "early infancy" OR pregnancy OR prenatal)  AND (probiotics OR "microecological agents" OR "live bacterial preparations" OR "probiotic formulations" OR synbiotics OR "beneficial bacteria" OR "beneficial microorganisms") AND ("allergic rhinitis" OR "atopic rhinitis" OR "perennial allergic rhinitis" OR "perennial rhinitis" OR "chronic allergic rhinitis" OR "chronic rhinitis" OR "atopic disease") | All Fields | 20 |
| Wanfang | (infant OR "early life" OR pregnancy) AND (probiotics OR Lactobacillus OR "Lactobacillus rhamnosus") AND ("allergic rhinitis" OR "atopic rhinitis" OR rhinitis OR "allergic diseases") | All Fields | 79 |
| CKNI | (infant OR "early life" OR pregnancy) AND (probiotics OR Lactobacillus OR "Lactobacillus rhamnosus") AND ("allergic rhinitis" OR "atopic rhinitis" OR rhinitis OR "allergic diseases") | All Fields | 44 |

| **Supplementary Table 2.** Subgroup analyses of AS in children aged > 1 year. | | | | |
| --- | --- | --- | --- | --- |
| Subgroup analysis | No. of studies | OR | 95% CI | P |
| Infant medication administration time |  | 0.87 | [0.76, 0.99] | 0.93 |
| ≤ 6month | 5 | 0.92 | [0.77, 1.10] | 0.34 |
| > 6month | 5 | 0.82 | [0.68, 0.99] | 0.04 |
| Target population for intervention |  | 0.87 | [0.76, 0.99] | 0.44 |
| Postpartum women and infant | 8 | 0.85 | [0.74, 0.98] | 0.03 |
| Infant | 2 | 1.00 | [0.69, 1.44] | 0.99 |
| Age at follow-up |  | 0.87 | [0.76, 0.99] | 0.67 |
| Early childhood | 3 | 0.86 | [0.60, 1.23] | 0.42 |
| Late childhood | 5 | 0.91 | [0.77, 1.07] | 0.24 |
| School age and adolescence | 3 | 0.93 | [0.60, 1.03] | 0.08 |
| Added prebiotic |  | 0.87 | [0.76, 0.99] | 0.89 |
| Yes | 2 | 0.88 | [0.71, 1.09] | 0.25 |
| No | 8 | 0.86 | [0.73, 1.02] | 0.08 |

Abbreviations: AS, atopic sensitization.
